# Impact of evidence-based nursing on clinical outcomes in patients with leukemia undergoing chemotherapy

**DOI:** 10.3389/fonc.2026.1858110

**Published:** 2026-08-03

**Authors:** Xiaoli Wang, Huijin Chen, Xinrong Zheng, Chengyuan Quan

**Affiliations:** 1 Department of Hematology and Oncology, Pingyang Hospital Affiliated to Wenzhou Medical University, Wenzhou, China; 2 Master of Nursing for the 2025 cohort, Macao Polytechnic University, Macau, China; 3 Department of General Practice and Geriatrics, Pingyang Hospital Affiliated to Wenzhou Medical University, Wenzhou, China; 4 Emergency Department, Pingyang Hospital Affiliated to Wenzhou Medical University, Wenzhou, China

**Keywords:** complications, evidence-based nursing, impact, leukemia, psychological state

## Abstract

**Purpose:**

This study aimed to evaluate whether evidence-based nursing interventions were associated with clinical outcomes in patients with leukemia undergoing chemotherapy and to support the development of optimized nursing strategies for hematologic oncology patients.

**Methods:**

This observational, single-center retrospective cohort study was conducted in the Pingyang Hospital Affiliated to Wenzhou Medical University, and was reported in accordance with the STROBE statement for observational studies. Adult patients with acute leukemia who underwent chemotherapy between February 2022 and February 2025 were screened using retrospective consecutive sampling according to predefined inclusion and exclusion criteria. Patients were classified according to the nursing model documented in electronic medical records: the conventional nursing group (n = 86) included patients treated from February 2022 to June 2023, when conventional nursing was the routine departmental nursing model, whereas the evidence-based nursing group (n = 91) included patients treated from July 2023 to February 2025, after a department-level evidence-based nursing pathway had been adopted in routine clinical practice. No patients were prospectively allocated to nursing interventions for the purpose of this study. Chemotherapy-related complications and SAS, SDS, ARMS, and SF-36 scores were extracted from medical records and routine nursing assessments. Separate binary logistic regression models were used to examine factors associated with infection, nausea/vomiting, and oral mucositis.

**Results:**

Compared with the Conventional group, the evidence-based group had lower incidences of infection (25.27% vs 41.86%, P = 0.019), nausea/vomiting (39.56% vs 65.12%, P < 0.001), and oral mucositis (17.58% vs 44.19%, P < 0.001). No significant between-group differences were observed for bleeding, bone marrow suppression, or phlebitis. Post-intervention SAS, SDS, and ARMS scores were lower in the evidence-based group, whereas SF-36 scores were statistically higher; however, the median between-group difference in SF-36 was modest (51.00 vs 47.50). In multivariable models, evidence-based nursing was associated with lower odds of infection (OR = 0.49, 95% CI: 0.25–0.96), nausea/vomiting (OR = 0.38, 95% CI: 0.20–0.73), and oral mucositis (OR = 0.31, 95% CI: 0.15–0.63).

**Conclusion:**

Compared with conventional care, evidence-based nursing was associated with lower rates of selected chemotherapy-related complications, lower anxiety and depression scores, better treatment adherence, and a modest increase in SF-36 scores among leukemia patients undergoing chemotherapy. Because this was a single-center retrospective before-after study, these findings should be interpreted as associations rather than definitive causal effects. Patients with poor ECOG status, abnormal WBC counts, or repeated chemotherapy cycles may require more intensive risk assessment and stratified nursing management.

## 1 Introduction

Leukemia is a clonal hematologic malignancy characterized by abnormal proliferation of hematopoietic cells and suppression of normal hematopoiesis (1). Acute leukemia, mainly including acute lymphoblastic leukemia and acute myeloid leukemia, is commonly accompanied by anemia, bleeding, infection, and organ infiltration, and often requires intensive chemotherapy and supportive care (2). During chemotherapy, patients with leukemia remain vulnerable to treatment-related adverse events, including myelosuppression, infection, bleeding, nausea/vomiting, and oral mucositis (3–5). These complications may increase symptom burden, interrupt treatment continuity, and place substantial demands on nursing care. In addition to physical complications, patients with leukemia frequently experience psychological distress, including anxiety and depression, which may be related to treatment adherence, symptom perception, and quality of life (6, 7). Therefore, nursing strategies that address both chemotherapy-related complications and patient-reported outcomes are clinically relevant in hematologic oncology.

Nursing care is an essential component of the multidisciplinary management of patients with leukemia undergoing chemotherapy. Conventional nursing usually focuses on routine monitoring, medication administration, basic health education, and general supportive care. However, patients undergoing chemotherapy often have complex and dynamic needs involving infection prevention, mucosal protection, symptom management, psychological support, treatment adherence, and self-monitoring. Evidence-based nursing emphasizes the integration of current research evidence, nurses’ clinical expertise, and patients’ preferences in nursing decision-making (8, 9). Recent studies have reported that evidence-based nursing or structured nursing interventions may be associated with better treatment adherence, quality of life, self-efficacy, and symptom-related outcomes in oncology or surgical populations (10, 11). In leukemia-related nursing research, recent studies have mainly focused on specific nursing models such as PDCA-based management or solution-focused nursing among patients undergoing chemotherapy (3, 12). These studies provide useful evidence for nursing practice but do not fully clarify the association between a comprehensive evidence-based nursing pathway and multiple clinical outcomes in leukemia patients receiving chemotherapy.

Accordingly, evidence-based nursing in leukemia patients undergoing chemotherapy remains insufficiently evaluated, particularly with respect to the simultaneous assessment of chemotherapy-related complications, psychological status, treatment adherence, and quality of life in routine clinical practice. In addition, different chemotherapy-related complications may have distinct nursing-sensitive components and clinical risk factors, which supports the need for outcome-specific analysis rather than relying only on a composite complication outcome. To address this knowledge gap, this retrospective cohort study evaluated whether evidence-based nursing was associated with clinical outcomes in patients with leukemia undergoing chemotherapy. We further explored factors associated with infection, nausea/vomiting, and oral mucositis to provide a basis for risk-stratified nursing management during chemotherapy.

## 2 Methods

### 2.1 Research design

This was a single-center retrospective cohort study conducted in the Hospital Affiliated to Wenzhou Medical University. The study was reported in accordance with the STROBE statement for observational studies. A retrospective consecutive sampling method was used, and all eligible patients during the study period were screened according to predefined inclusion and exclusion criteria. Adult patients with acute leukemia who underwent chemotherapy between February 2022 and February 2025 were screened from the electronic medical record system and nursing documentation system according to predefined inclusion and exclusion criteria. Patients were classified according to the nursing model recorded during hospitalization. The Conventional group included patients treated from February 2022 to June 2023, when conventional nursing was the routine departmental nursing model, whereas the evidence-based group included patients treated from July 2023 to February 2025, after a department-level evidence-based nursing pathway had been adopted in routine clinical practice. The research team did not prospectively assign patients to either nursing model, recruit participants for an intervention, or alter clinical care; all group classifications and outcome assessments were based on retrospectively extracted medical and nursing records. A total of 177 patients were included, comprising 86 in the Conventional group and 91 in the evidence-based group.

This study adhered to the ethical principles of the Declaration of Helsinki and was reviewed and approved by the hospital’s ethics committee. Due to the retrospective design and use of anonymized medical and nursing records, the requirement for informed consent was waived by the ethics committee. The patient information was strictly anonymized during the data collection and analysis process. All study data were sourced from the hospital’s electronic medical record (EMR) system and nursing documentation system. During data extraction, patient names, home addresses, and other personally identifiable information were removed. The research data were managed with unique coding for numbering.

### 1.2 Inclusion and exclusion criteria

Inclusion criteria: (1) Confirmed diagnosis of acute lymphoblastic leukemia (ALL) or acute myeloid leukemia (AML) by bone marrow aspiration and immunophenotyping; (2) Age range of 18–75 years; (3) Received standardized chemotherapy regimen; (4) Estimated survival duration ≥3 months; (5) Complete medical records, including nursing documentation, laboratory test results, and outcome data.

Exclusion criteria: (1) Patients with severe cardiac, hepatic, or renal dysfunction (Child-Pugh Class C or NYHA Class IV); (2) Patients with severe infection or active bleeding at initial diagnosis; (3) Patients with concurrent other malignancies; (4) Incomplete nursing records that preclude determination of nursing model classification; (5) Incomplete data due to department transfer or voluntary discharge during hospitalization.

### 1.3 Nursing interventions

This section summarizes the nursing protocols that had already been used in routine clinical practice before retrospective data extraction. This was an observational rather than interventional study; the nursing protocols were not prospectively assigned or modified for the purpose of this study. The evidence-based nursing pathway was delivered throughout each chemotherapy hospitalization, from admission to discharge. Routine monitoring was performed during nursing rounds, health education was reinforced during hospitalization and before discharge, and psychological and scale assessments were recorded at routine departmental assessment time points. Group classification was based on existing nursing documentation and electronic medical records.

#### 1.3.1 Conventional group

During the conventional nursing period, patients in the Conventional group received conventional nursing, including admission education, vital sign and laboratory monitoring, chemotherapy-related observation, basic oral, skin, and dietary care, and discharge instructions.

#### 1.3.2 Evidence-based group

During the evidence-based nursing period, the department used an evidence-based nursing pathway developed according to the JBI (Joanna Briggs Institute) model of evidence-based healthcare (13), in addition to conventional nursing. According to nursing documentation, the pathway included evidence-based question development, evidence retrieval, evidence appraisal and integration, pathway use, and documentation/quality evaluation. The evidence-based question was developed using the PICO framework: patients with leukemia undergoing chemotherapy, evidence-based nursing, conventional nursing care, and outcomes including complications, psychological status, treatment adherence, and quality of life.

Evidence retrieval: PubMed, Cochrane Library, CINAHL, Embase, CNKI, and Wanfang were searched for evidence-based guidelines, systematic reviews, and randomized controlled trials related to chemotherapy nursing for leukemia. The search period was January 2019 to July 2023, and the language was limited to English and Chinese. Evidence appraisal and integration were performed independently by two nurses trained in evidence-based nursing, and the evidence was classified according to the JBI evidence hierarchy.

The main nursing components were organized into infection prevention, chemotherapy adverse reaction management, psychological support, and health education. Detailed procedural parameters are summarized in **Supplementary Table 1**. (a) Infection prevention: environmental management, oral care, perianal care, skin care, PICC-site monitoring, and education regarding infection warning signs were provided according to departmental nursing procedures. (b) Chemotherapy adverse reaction management: nurses monitored symptoms related to nausea/vomiting, oral mucositis, bleeding, myelosuppression, phlebitis, and other chemotherapy-related adverse reactions; provided dietary guidance and non-pharmacological support; and coordinated physician-prescribed supportive care when needed. (c) Psychological support: routine psychological assessment, therapeutic communication, cognitive-behavioral support, peer support, and family involvement were provided according to patients’ documented needs. (d) Health education: nurses provided disease education, medication-related education, self-monitoring guidance, and discharge instructions during hospitalization and before discharge.

Medication-related supportive measures, including antiemetic administration and G-CSF use, were prescribed by physicians and implemented according to departmental supportive-care protocols; these measures were not used to define group allocation. Implementation of the pathway and routine assessment results were recorded in nursing documentation, and no additional intervention was introduced by the research team.

### 1.4 Evaluation indicators

All outcome data in this study were obtained from the hospital electronic medical record system and routine health assessment records of the departments. Patients completed scale assessments before and after the corresponding nursing period according to the department’s routine management process. For patients who attended scheduled outpatient follow-up visits, the scales were completed under the guidance of outpatient nursing staff during the consultation. For those who failed to attend follow-up, nursing staff obtained the scale information via telephone communication and recorded it accordingly. Telephone supplementation was used only for patient-reported scale outcomes, including Self-Rating Anxiety Scale (SAS) (14), Self-Rating Depression Scale (SDS) (15), The Adherence to Refills and Medications Scale (ARMS) (16), and The Short Form Health Survey (17); complication outcomes were extracted from electronic medical records and nursing documentation. To reduce information bias, telephone follow-up was conducted using the same scale items and scoring criteria as those used during outpatient assessment. Nursing staff read each item in a standardized manner and recorded the patients’ responses directly, without providing additional explanations or leading prompts. All assessments were conducted by nursing staff who had received standardized training on the scale structure, scoring methodology, and interview protocols to ensure consistency in evaluation. The evaluators did not participate in the implementation of evidence-based nursing interventions to minimize assessment bias. After obtaining ethical approval, the research team retrospectively extracted relevant data from previous electronic medical records (EMRs) and nursing documentation. Data were organized through independent dual-entry and cross-verification to ensure accuracy and completeness.

#### 1.4.1 Primary outcomes

Complication occurrence: Record the occurrence of complications during chemotherapy, including infections (oral infections, respiratory infections, perianal infections, etc.), bleeding, nausea and vomiting, oral mucositis, phlebitis, and bone marrow suppression.

#### 1.4.2 Secondary outcomes

1. Psychological status: SAS score and SDS score were used to assess the patients’ psychological status. The SAS and SDS each contain 20 items, using a 4-point Likert scale, with the standard score calculated as the gross score multiplied by 1.25.The SAS scale scores of 50–59 indicate mild anxiety, 60–69 indicate moderate anxiety, and ≥70 indicate severe anxiety; the SDS scale scores of 53–62 indicate mild depression, 63–72 indicate moderate depression, and ≥73 indicate severe depression. The evaluation time point was the same as the primary outcome. The scale has good reliability and validity in leukemia patients.
2. Treatment adherence: ARMS was used to assess patient compliance. This scale consists of 12 items and is primarily used to assess patients’ adherence to medication and drug supplementation. Each entry uses a 4-level rating scale (1=never, 2=sometimes, 3=often, 4=always), with a total score range of 12–48 points. A lower total score on the scale indicates better treatment adherence, while a higher score suggests poorer adherence.
3. Quality of life: SF-36 was used to assess patients’ quality of life. The scale comprises 8 dimensions (physiological function, physiological capacity, somatic pain, general health, vitality, social function, emotional function, and mental health), with each dimension scored on a 0–100 scale. Higher scores indicate better quality of life.

### 1.5 Statistical methods

SPSS 26.0 software was used for data analysis. For normally distributed measurement data, the mean ± standard deviation (*x̄* ± *s*) was used, and the t-test was employed for comparisons. For non-normally distributed measurement data, the median (interquartile range) [M(Q_1_, Q_3_)] was reported, and the Mann-Whitney U test was utilized for intergroup comparisons. Count data were expressed as frequencies (percentages) [n (%)], and intergroup comparisons were performed using the χ² test or Fisher’s exact test. Logistic regression analysis was used to identify the independent risk factors for complications, with the calculation of odds ratio (OR) and 95% confidence interval (CI). P<0.05 was considered statistically significant.

## 2 Results

### 2.1 Baseline data

A total of 177 leukemia patients undergoing chemotherapy were included in the analysis, with 86 patients in the conventional nursing group and 91 patients in the evidence-based nursing group. Baseline characteristics are summarized in **Table 1**. The two groups were generally comparable in general demographic characteristics, disease-related characteristics and laboratory indicators. Age, BMI, sex distribution, education level, marital status, hospital stay, time to chemotherapy, smoking history, diabetes, and hypertension were comparable between the two groups (all P > 0.05; **Table 1**).

**Table 1:** Baseline data.

Variables	Total (n = 177)	Conventional group (n = 86)	Evidence-based group(n = 91)	Statistic	P
Age, (years), M (Q_1_, Q_3_)	50.00 (43.00, 59.00)	50.50 (43.00, 59.75)	50.00 (43.00, 58.00)	Z=-0.38	0.702
Gender, n(%)				χ²=0.01	0.942
Female	88 (49.72)	43 (50.00)	45 (49.45)		
Male	89 (50.28)	43 (50.00)	46 (50.55)		
BMI, (kg/m2), M (Q_1_, Q_3_)	22.30 (20.90, 23.50)	22.35 (20.95, 23.50)	22.20 (20.95, 23.40)	Z=-0.39	0.699
Education, n(%)				χ²=0.05	0.978
College	59 (33.33)	28 (32.56)	31 (34.07)		
High school	59 (33.33)	29 (33.72)	30 (32.97)		
Junior high	59 (33.33)	29 (33.72)	30 (32.97)		
Marital Status, n(%)				χ²=1.44	0.696
Divorced	16 (9.04)	6 (6.98)	10 (10.99)		
Married	100 (56.50)	52 (60.47)	48 (52.75)		
Single	41 (23.16)	19 (22.09)	22 (24.18)		
Widowed	20 (11.30)	9 (10.47)	11 (12.09)		
Hospital days, (d), Mean ± SD	30.23 ± 8.62	30.22 ± 7.41	30.24 ± 9.67	t=-0.02	0.987
Time to Chemo days, (d), M (Q_1_, Q_3_)	5.00 (4.00, 6.00)	5.00 (4.00, 6.00)	5.00 (4.00, 6.00)	Z=-0.47	0.640
Smoking History, n(%)				χ²=0.10	0.754
No	134 (75.71)	66 (76.74)	68 (74.73)		
Yes	43 (24.29)	20 (23.26)	23 (25.27)		
Diabetes, n(%)				χ²=0.57	0.449
No	142 (80.23)	71 (82.56)	71 (78.02)		
Yes	35 (19.77)	15 (17.44)	20 (21.98)		
Hypertension, n(%)				χ²=0.04	0.848
No	114 (64.41)	56 (65.12)	58 (63.74)		
Yes	63 (35.59)	30 (34.88)	33 (36.26)		
Leukemia Type, n(%)				χ²=0.15	0.695
ALL	45 (25.42)	23 (26.74)	22 (24.18)		
AML	132 (74.58)	63 (73.26)	69 (75.82)		
Cytogenetic Risk, n(%)				χ²=1.09	0.581
Adverse	26 (14.69)	15 (17.44)	11 (12.09)		
Favorable	42 (23.73)	19 (22.09)	23 (25.27)		
Intermediate	109 (61.58)	52 (60.47)	57 (62.64)		
ECOG Score, n(%)				χ²=0.13	0.714
0-1	117 (66.10)	58 (67.44)	59 (64.84)		
2-3	60 (33.90)	28 (32.56)	32 (35.16)		
Chemotherapy Regimen, n(%)				χ²=0.16	0.924
DA	57 (32.20)	27 (31.40)	30 (32.97)		
Hyper-CVAD	45 (25.42)	23 (26.74)	22 (24.18)		
IA	75 (42.37)	36 (41.86)	39 (42.86)		
Hemoglobin, (g/L), Mean ± SD	97.02 ± 12.01	96.43 ± 12.46	97.57 ± 11.61	t=-0.63	0.529
Platelet Count, (×10^9^/L), Mean ± SD	85.71 ± 15.88	83.59 ± 15.27	87.71 ± 16.26	t=-1.74	0.084
WBC count, (×10^9^/L), M (Q_1_, Q_3_)	12.40 (7.30, 22.80)	13.15 (6.90, 24.23)	12.00 (7.75, 18.15)	Z=-0.20	0.844
Chemotherapy Cycles, M (Q_1_, Q_3_)	4.00 (3.00, 4.00)	4.00 (3.00, 4.00)	4.00 (3.00, 4.50)	Z=-0.93	0.350

BMI, body mass index; ALL, acute lymphoblastic leukemia; AML, acute myeloid leukemia; ECOG, Eastern Cooperative Oncology Group; DA, daunorubicin plus cytarabine; Hyper-CVAD, hyperfractionated cyclophosphamide, vincristine, doxorubicin, and dexamethasone; IA, idarubicin plus cytarabine; WBC, white blood cell.

Leukemia type, cytogenetic risk, ECOG score, chemotherapy regimen, hemoglobin, platelet count, WBC count, and chemotherapy cycles did not differ significantly between the two groups (all P > 0.05; **Table 1**). Overall, the baseline demographic, clinical, and laboratory characteristics were comparable between the two groups (**Table 1**).

### 2.2 Complications occurrence

After baseline comparability was assessed, chemotherapy-related complications were compared between the two groups, as shown in **Table 2**.

**Table 2:** Complications.

Variables	Total (n = 177)	Conventional group (n = 86)	Evidence-based group(n = 91)	Statistic	P
Infection, n(%)	59 (33.33)	36 (41.86)	23 (25.27)	χ²=5.47	0.019
Bleeding, n(%)	33 (18.64)	19 (22.09)	14 (15.38)	χ²=1.31	0.252
Nausea Vomiting, n(%)	92 (51.98)	56 (65.12)	36 (39.56)	χ²=11.57	<0.001
Oral Mucositis, n(%)	54 (30.51)	38 (44.19)	16 (17.58)	χ²=14.76	<0.001
Phlebitis, n(%)	28 (15.82)	18 (20.93)	10 (10.99)	χ²=3.28	0.070
Bone Marrow Suppression, n(%)	47 (26.55)	26 (30.23)	21 (23.08)	χ²=1.16	0.281

Compared with the conventional nursing group, the evidence-based nursing group showed lower rates of infection, nausea/vomiting, and oral mucositis, whereas bleeding, bone marrow suppression, and phlebitis did not differ significantly between groups (**Table 2**).

### 2.3 Psychological state

**Figure 1:**
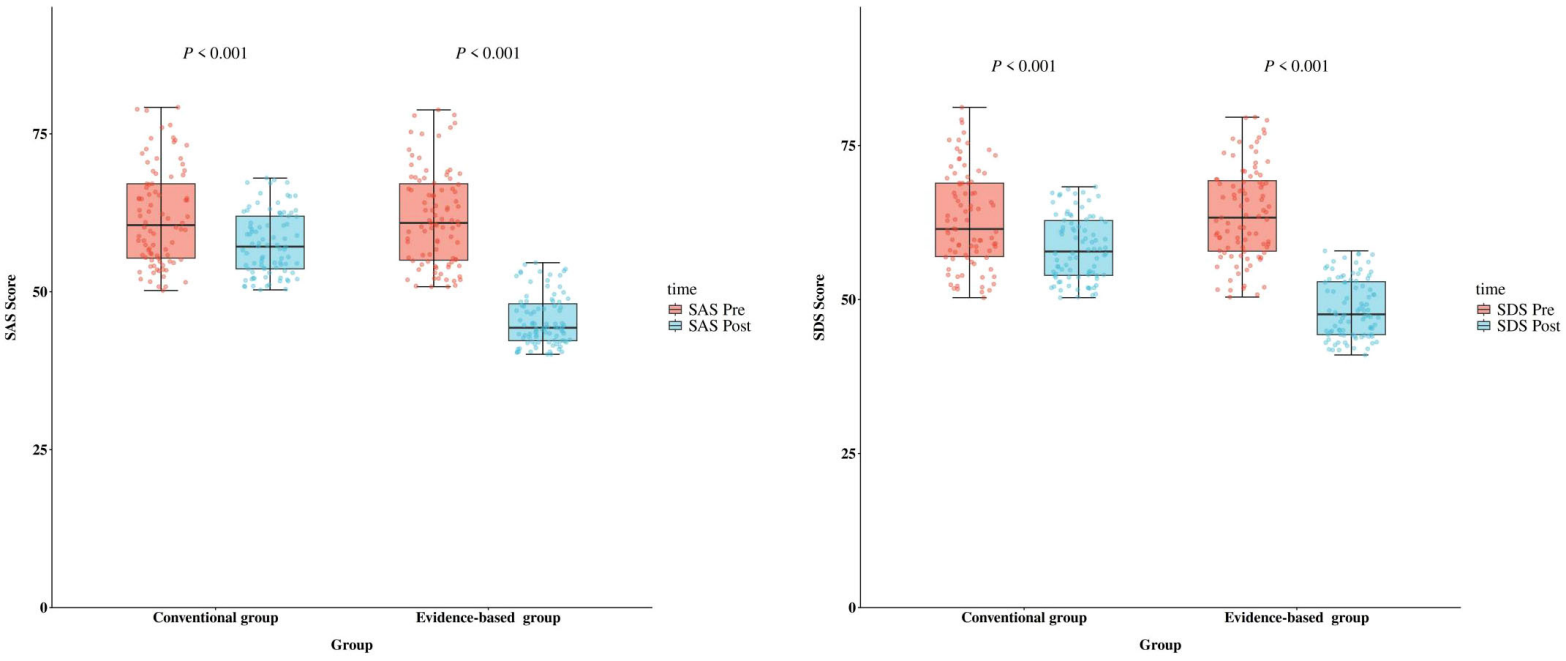
Pre- and post-intervention SAS and SDS score distributions. P values above each group indicate within-group pre-post comparisons.

Pre-intervention SAS and SDS scores were comparable between groups, whereas post-intervention SAS and SDS scores were lower in the evidence-based nursing group than in the conventional nursing group (**Table 3**; **Figure 1**).

**Table 3:** Psychological status.

Variables	Total (n = 177)	Conventional group (n = 86)	Evidence-based group (n = 91)	Statistic	P
SAS Pre, M (Q_1_, Q_3_)	60.90 (55.10, 67.10)	60.55 (55.32, 67.10)	60.90 (55.00, 67.10)	Z=-0.14	0.891
SAS Post, M (Q_1_, Q_3_)	51.70 (44.30, 56.80)	57.15 (53.62, 61.98)	44.30 (42.25, 48.10)	Z=-10.80	<0.001
SDS Pre, M (Q_1_, Q_3_)	62.80 (57.60, 69.10)	61.45 (56.97, 68.90)	63.30 (57.85, 69.30)	Z=-0.78	0.433
SDS Post, M (Q_1_, Q_3_)	53.40 (47.50, 57.50)	57.80 (53.92, 62.82)	47.60 (44.30, 52.90)	Z=-9.31	<0.001

SAS, Self-Rating Anxiety Scale; SDS, Self-Rating Depression Scale.

### 2.4 Treatment compliance

**Figure 2:**
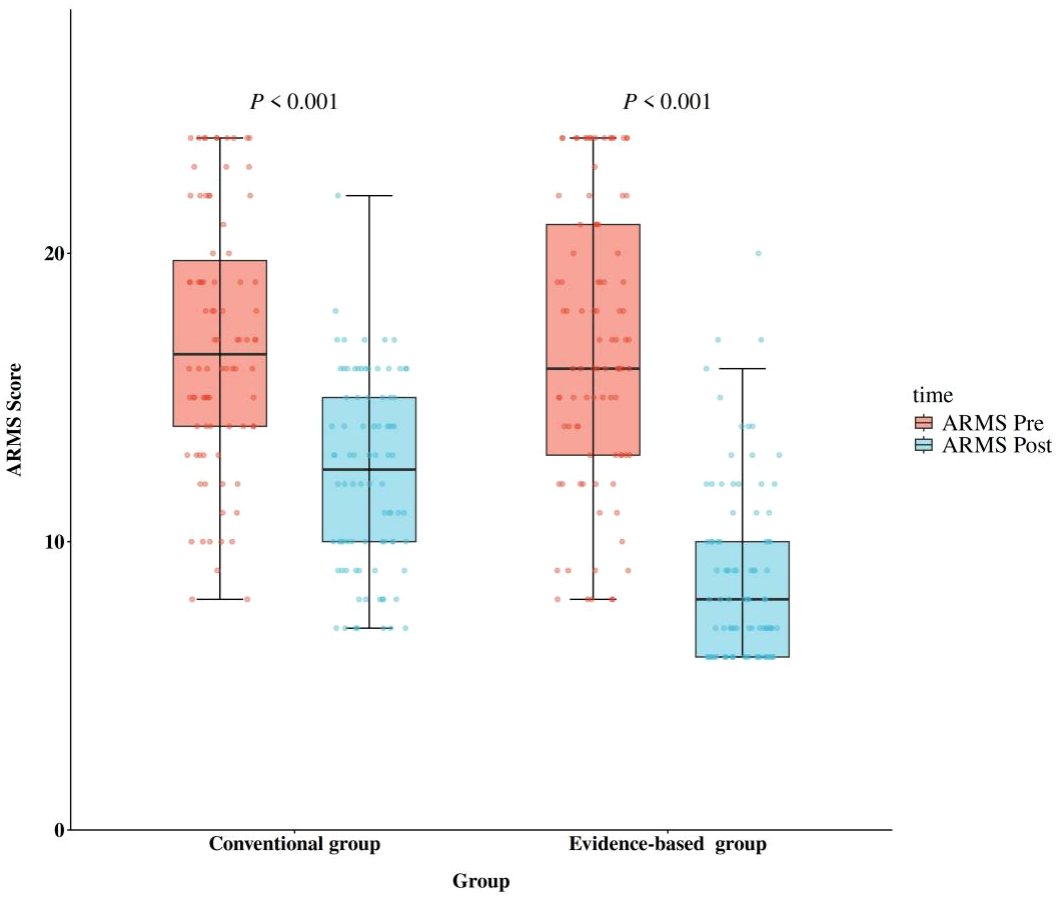
Pre- and post-intervention ARMS score distributions. P values above each group indicate within-group pre-post comparisons.

Pre-intervention ARMS scores were comparable between groups, whereas post-intervention ARMS scores were lower in the evidence-based nursing group than in the conventional nursing group (**Table 4**; **Figure 2**). Because lower ARMS scores indicate better adherence, the post-intervention results suggest better adherence-related scores in the evidence-based nursing group.

**Table 4:** Treatment compliance.

Variables	Total (n = 177)	Conventional group (n = 86)	Evidence-based group (n = 91)	Statistic	P
ARMS Pre, M (Q_1_, Q_3_)	16.00 (14.00, 21.00)	16.50 (14.00, 19.75)	16.00 (13.00, 21.00)	Z=-0.07	0.945
ARMS Post, M (Q_1_, Q_3_)	10.00 (7.00, 13.00)	12.50 (10.00, 15.00)	8.00 (6.00, 10.00)	Z=-6.86	<0.001

ARMS, Adherence to Refills and Medications Scale.

### 2.5 Quality of life

**Figure 3:**
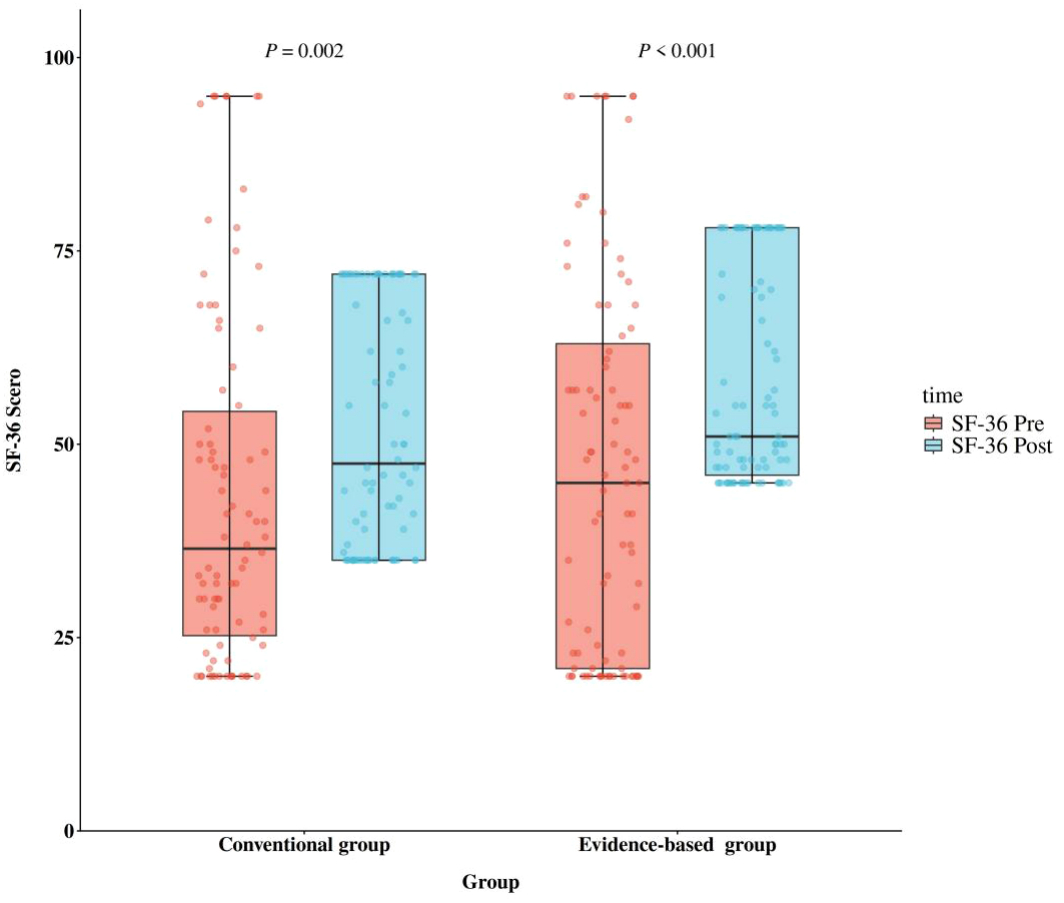
Pre- and post-intervention SF-36 score distributions. P values above each group indicate within-group pre-post comparisons.

Pre-intervention SF-36 scores were comparable between groups, whereas post-intervention SF-36 scores were statistically higher in the evidence-based nursing group than in the conventional nursing group (**Table 5**; **Figure 3**). However, the absolute median difference between groups was small. Because a minimally clinically important difference threshold for the SF-36 total score was not prespecified in this retrospective study, the clinical relevance of this finding should not be overinterpreted.

**Table 5:** Quality of life.

Variables	Total (n = 177)	Conventional group (n = 86)	Evidence-based group (n = 91)	Statistic	P
SF-36 Pre, M (Q_1_, Q_3_)	41.00 (23.00, 60.00)	36.50 (25.25, 54.25)	45.00 (21.00, 63.00)	Z=-0.71	0.479
SF-36 Post, M (Q_1_, Q_3_)	50.00 (45.00, 72.00)	47.50 (35.00, 72.00)	51.00 (46.00, 78.00)	Z=-3.53	<0.001

SF-36, Short Form Health Survey.

### 2.6 Logistic regression analyses for infection, nausea/vomiting, and oral mucositis

Based on the between-group comparisons, separate binary logistic regression models were constructed for infection, nausea/vomiting, and oral mucositis to further examine factors associated with these outcomes. Nursing approach (Evidence-based=0, conventional nursing=1), cytogenetic risk (Favorable=0, Intermediate=1, Adverse=2), ECOG score(0–1 score=0, 1-2=-1score=1), WBC count, number of chemotherapy cycles, and chemotherapy regimen(DA = 0, Hyper-CVAD=1, IA = 2) were included as candidate variables.

For infection, the multivariable model showed that nursing approach, ECOG score, WBC count, and number of chemotherapy cycles were associated with infection occurrence, whereas cytogenetic risk and chemotherapy regimen were not statistically significant after adjustment (**Table 6**).

**Table 6:** Univariate and multivariable logistic regression analyses for infection.

Variable	Univariate OR	95% CI	P	Multivariate OR	95% CI	P
Nursing approach	0.47	0.25–0.88	0.019	0.49	0.25–0.96	0.037
Cytogenetic risk	1.86	1.05–3.31	0.034	1.74	0.94–3.22	0.078
ECOG score	2.18	1.17–4.06	0.014	1.93	1.00–3.72	0.049
WBC count	1.42	1.10–1.84	0.007	1.36	1.04–1.79	0.026
Chemotherapy cycles	1.68	1.12–2.51	0.012	1.54	1.01–2.36	0.046
Chemotherapy regimen	1.31	0.72–2.39	0.374	1.22	0.64–2.33	0.548

OR, odds ratio; CI, confidence interval; ECOG, Eastern Cooperative Oncology Group; WBC, white blood cell.

For nausea/vomiting, nursing approach and number of chemotherapy cycles were associated with the outcome after adjustment, whereas cytogenetic risk, ECOG score, WBC count, and chemotherapy regimen were not statistically significant in the multivariable model (**Table 7**).

**Table 7:** Univariate and multivariable logistic regression analyses for nausea/vomiting.

Variable	Univariate OR	95% CI	P	Multivariate OR	95% CI	P
Nursing approach	0.35	0.19–0.65	<0.001	0.38	0.20–0.73	0.004
Cytogenetic risk	1.42	0.82–2.47	0.213	1.31	0.72–2.40	0.374
ECOG score	1.76	0.96–3.22	0.067	1.61	0.85–3.07	0.145
WBC count	1.21	0.95–1.55	0.121	1.16	0.89–1.51	0.271
Chemotherapy cycles	1.89	1.26–2.84	0.002	1.75	1.13–2.70	0.012
Chemotherapy regimen	1.58	0.88–2.82	0.124	1.46	0.78–2.74	0.238

OR, odds ratio; CI, confidence interval; ECOG, Eastern Cooperative Oncology Group; WBC, white blood cell.

For oral mucositis, nursing approach and number of chemotherapy cycles were associated with the outcome after adjustment, whereas cytogenetic risk, ECOG score, WBC count, and chemotherapy regimen were not statistically significant in the multivariable model (**Table 8**).

**Table 8:** Univariate and multivariable logistic regression analyses for oral mucositis.

Variable	Univariate OR	95% CI	P	Multivariate OR	95% CI	P
Nursing approach	0.27	0.14–0.53	<0.001	0.31	0.15–0.63	0.001
Cytogenetic risk	1.69	0.94–3.05	0.079	1.58	0.84–2.98	0.156
ECOG score	1.84	0.97–3.47	0.061	1.66	0.84–3.29	0.145
WBC count	1.29	1.01–1.65	0.043	1.24	0.95–1.62	0.109
Chemotherapy cycles	2.03	1.33–3.10	0.001	1.86	1.18–2.93	0.008
Chemotherapy regimen	1.47	0.80–2.72	0.216	1.35	0.70–2.61	0.368

OR, odds ratio; CI, confidence interval; ECOG, Eastern Cooperative Oncology Group; WBC, white blood cell.

Overall, nursing approach and chemotherapy cycles were consistently associated with infection, nausea/vomiting, and oral mucositis in the adjusted models, while ECOG score and WBC count were additionally associated with infection.

## 3 Discussion

Patients with leukemia often enter a phase of myelosuppression and neutropenia after chemotherapy, during which the mucosal barrier is compromised and immune defense capacity is reduced, significantly increasing the risk of infection. Infection remains one of the major treatment-related adverse outcomes in patients with hematologic malignancies (17, 18). The present findings highlight infection, nausea/vomiting, and oral mucositis as major nursing-sensitive outcomes that warrant focused monitoring during chemotherapy. The absence of clear between-group differences in bleeding, bone marrow suppression, and phlebitis suggests that these outcomes may be more strongly influenced by disease burden, chemotherapy intensity, hematopoietic reserve, vascular condition, and infusion-related factors. These findings suggest that evidence-based nursing may be more closely associated with nursing-sensitive outcomes such as symptom management, mucosal barrier protection, and early risk warning. One possible explanation is that a structured nursing pathway may improve the consistency of infection prevention, mucosal care, antiemetic management, symptom monitoring, and patient education, which may support earlier recognition and management of nursing-sensitive adverse events. By contrast, bleeding, bone marrow suppression, and phlebitis are likely to be influenced by disease burden, chemotherapy intensity, hematopoietic reserve, vascular condition, and infusion-related factors, no statistically significant differences were observed under the sample size conditions of this study. These findings are broadly consistent with those of Han and Tian (19) and Zhang et al. (20), who reported favorable associations between structured or preventive nursing strategies and complication-related or psychological outcomes in patients with leukemia undergoing chemotherapy. In the present study, separate logistic regression models were constructed for infection, nausea/vomiting, and oral mucositis, rather than using a composite complication outcome. This approach provides a more clinically interpretable analysis because different chemotherapy-related complications may have distinct mechanisms and nursing-sensitive components. The outcome-specific models suggest that the associations differed across complications. Nursing approach and chemotherapy cycles were consistently related to the three nursing-sensitive complications, whereas ECOG score and WBC count appeared more specifically related to infection. This result suggests that the complications are not solely determined by the nursing model, but rather result from the combined effects of disease biological aggressiveness, baseline physical condition, tumor burden/inflammatory status, and cumulative treatment exposure. High cytogenetic risk and poor ECOG scores often indicate more severe disease, poorer tolerance, and higher risks of infections and other adverse outcomes; abnormal WBC counts and increased chemotherapy cycles may reflect higher disease activity and greater cumulative bone marrow toxicity (21). Based on this, the value of evidence-based nursing lies not only in the overall improvement of outcomes, but also in providing a practical risk management framework for high-risk patients. For patients with high-risk cytogenetics, poor physical condition, or those undergoing multiple cycles of chemotherapy, it is essential to advance and strengthen evidence-based nursing, establish dynamic evaluation and timely adjustment mechanisms, and achieve stratified individualized nursing management (22).

Regarding psychological status, the observed between-group differences suggest that structured nursing care may be associated with lower emotional distress during chemotherapy. Patients with leukemia experience rapid disease progression, prolonged treatment cycles, and multiple complications. During hospitalization for chemotherapy, they face multiple pressures including uncertain prognosis, restricted social roles, and financial burdens, with the incidence of negative emotions such as anxiety and depression reaching as high as 50% (23). A review by Kuczmarski et al. (24) indicated that patients with hematologic malignancies are prone to depression throughout the disease course. Depression not only reduces quality of life but is also associated with more treatment-related complications and poorer survival outcomes. Therefore, systematic screening and psychological intervention should be strengthened. A randomized controlled trial by El-Jawahri (25) et al. in AML patients also demonstrated that early integration of supportive/palliative care into intensive chemotherapy significantly improved psychological outcomes such as quality of life, anxiety, and depression. The psychological assessment, therapeutic communication, cognitive behavioral techniques, peer support, and family involvement in this evidence-based nursing program essentially constitute a comprehensive psychological support chain. This approach helps patients reduce disease uncertainty, improve negative automatic thinking, and enhance treatment control, which is consistent with previous research findings.

For treatment adherence, the ARMS findings suggest a favorable association between structured nursing care and adherence-related behaviors. Treatment adherence is a critical factor influencing the efficacy of chemotherapy. Poor adherence may lead to treatment discontinuation or dose adjustment, thereby compromising therapeutic outcomes. For leukemia patients undergoing chemotherapy, adherence is not only reflected in timely receipt of chemotherapy and adjuvant therapy, but also includes the implementation of infection prevention, oral care, dietary management, recognition of abnormal symptoms, and follow-up scheduling. Evidence-based nursing, through disease education, risk communication, medication reminders, family collaboration, and self-monitoring training, can enhance patients’ understanding of the necessity of treatment, reduce negative coping behaviors caused by fear, misunderstanding, or insufficient information, thereby improving treatment adherence. Li (12) and Zhang et al. (8) reported favorable relationships between evidence-based nursing and adherence, quality of life, or self-efficacy in other clinical populations. The findings of this study are consistent with the aforementioned research, supporting a possible beneficial association between evidence-based nursing and treatment adherence among leukemia patients undergoing chemotherapy.

In terms of quality of life, the evidence-based group showed a statistically higher post-intervention SF-36 score than the Conventional group. For leukemia patients, quality of life is influenced by multiple factors, including physiological symptoms, physical decline, psychological burden, impaired social functioning, and treatment uncertainty. Therefore, single-dimensional interventions often fail to achieve optimal outcomes (26). In this study, evidence-based nursing comprehensively covered infection prevention, adverse reaction management, psychological support, and health education, which may partly explain the favorable trend in SF-36 scores. However, the absolute median difference between the two groups was small, and this study did not prespecify a minimally clinically important difference threshold for the SF-36 total score in leukemia patients undergoing chemotherapy. Therefore, although the statistical result suggests a favorable association between evidence-based nursing and patient-reported quality of life, the clinical magnitude of this improvement should not be overinterpreted. Previous studies have reported favorable relationships between evidence-based nursing or integrated supportive care and quality-of-life-related outcomes, psychological symptoms, symptom burden, and self-efficacy (27). Future prospective studies should incorporate prespecified clinically meaningful thresholds or disease-specific quality-of-life instruments to determine whether the observed SF-36 changes translate into meaningful improvements from the patient perspective.

Several limitations should be considered. Because the conventional care and evidence-based groups were enrolled in two consecutive periods, this study had an inherent before-after structure, and time-trend bias could not be fully excluded. Baseline characteristics were compared and relevant clinical factors were adjusted in multivariable analysis; however, these measures can only reduce measured imbalance and cannot control unrecorded period-related changes. The retrospective dataset did not allow a complete quantitative assessment of whether supportive treatment, anti-infective strategies, antiemetic prescribing, clinical workflow, or nursing staffing intensity changed across the study period; therefore, residual time-trend bias remains possible. Partial results were derived from previous scale records and telephone follow-up supplements. Although standardized training and double-checking were implemented during the study to minimize bias, the proportions, timing, and outcome-specific distribution of telephone versus outpatient scale completion were not prospectively balanced or fully auditable; differential information bias for subjective outcomes such as SAS, SDS, ARMS, and SF-36 therefore remains possible. This study primarily focuses on outcomes during and shortly after hospitalization for chemotherapy, without evaluating long-term follow-up outcomes such as readmission, recurrent infections, persistent psychological burden, and changes in long-term quality of life. Future multicenter prospective studies with standardized complication definitions and randomized or quasi-experimental designs are needed to confirm these findings and clarify the relative contribution of different evidence-based nursing components.

## 4 Conclusion

In this single-center retrospective before-after study, evidence-based nursing was associated with lower rates of selected chemotherapy-related complications, improved emotional-status scores, better treatment adherence, and a modest increase in SF-36 scores among leukemia patients undergoing chemotherapy. These findings support further prospective evaluation and cautious clinical application of a structured nursing pathway centered on infection prevention, symptom control, psychological support, and health education, rather than proving a definitive causal effect. Based on the separate multivariable models, patients with poor ECOG status, abnormal WBC counts, or repeated chemotherapy cycles may require more intensive monitoring and stratified nursing management, particularly for infection, nausea/vomiting, and oral mucositis.

## Data Availability

The original contributions presented in the study are included in the article/**Supplementary Material**. Further inquiries can be directed to the corresponding author.
