# Supplementary material for: Impact of evidence-based nursing on clinical outcomes in patients with leukemia undergoing chemotherapy
Source: Front Oncol. 2026 Aug 3;16:1858110. doi: 10.3389/fonc.2026.1858110 (PMC13477912; doi:10.3389/fonc.2026.1858110)
Supplement: Supplementary file 1 [file Table1.docx]

**Supplementary Table S1. Procedural details of the evidence-based nursing pathway**

| Component | Procedural details moved from the main text |
| --- | --- |
| Environmental management | Laminar flow ward or single-room placement when available; ultraviolet disinfection twice daily for 30 minutes; visitor restriction; visitors wearing masks and shoe covers. |
| Oral care | Modified Bass brushing with a soft-bristled toothbrush three times daily; mouthwash selected according to oral pH: 2% sodium bicarbonate for pH < 6.5, 2%–3% boric acid for pH > 7.0, and normal saline for pH 6.5–7.0; rinsing after meals and before bedtime; oral cryotherapy with ice chips for 5–10 minutes during chemotherapy when clinically appropriate. |
| Perianal care | Guidance on regular bowel movements; perianal cleansing after defecation and before bedtime; 1:5000 potassium permanganate solution used when clinically appropriate and recorded in nursing documentation. |
| Skin and PICC-site care | Skin hygiene and dryness maintenance; warm-water sponge bathing when needed; PICC-site dressing care twice weekly; monitoring of puncture-site condition. |
| Nausea/vomiting management | Antiemetics administered according to physicians’ orders before chemotherapy; relaxation training, music therapy, small frequent meals, and avoidance of greasy or spicy foods. |
| Myelosuppression-related nursing | CBC monitoring; protective isolation according to WBC/neutrophil count and departmental protocol; G-CSF administered according to physicians’ orders; activity restriction and bleeding prevention according to platelet count and clinical condition. |
| Bleeding prevention | Use of soft-bristled toothbrushes; prevention of nasal dryness; avoidance of unnecessary intramuscular injections; gentle nursing procedures. |
| Psychological support and health education | SAS/SDS-based assessment; therapeutic communication; cognitive-behavioral support; peer support; family education; disease education; medication-related education; self-monitoring of infection and bleeding signs. |
